# Supplementary figures and images for: Short- and mid-term temporal variability of the human urinary microbiota: a prospective observational cohort study
Source: BMC Microbiol. 2025 Apr 16;25:222. doi: 10.1186/s12866-025-03915-7 (PMC12001408; doi:10.1186/s12866-025-03915-7)

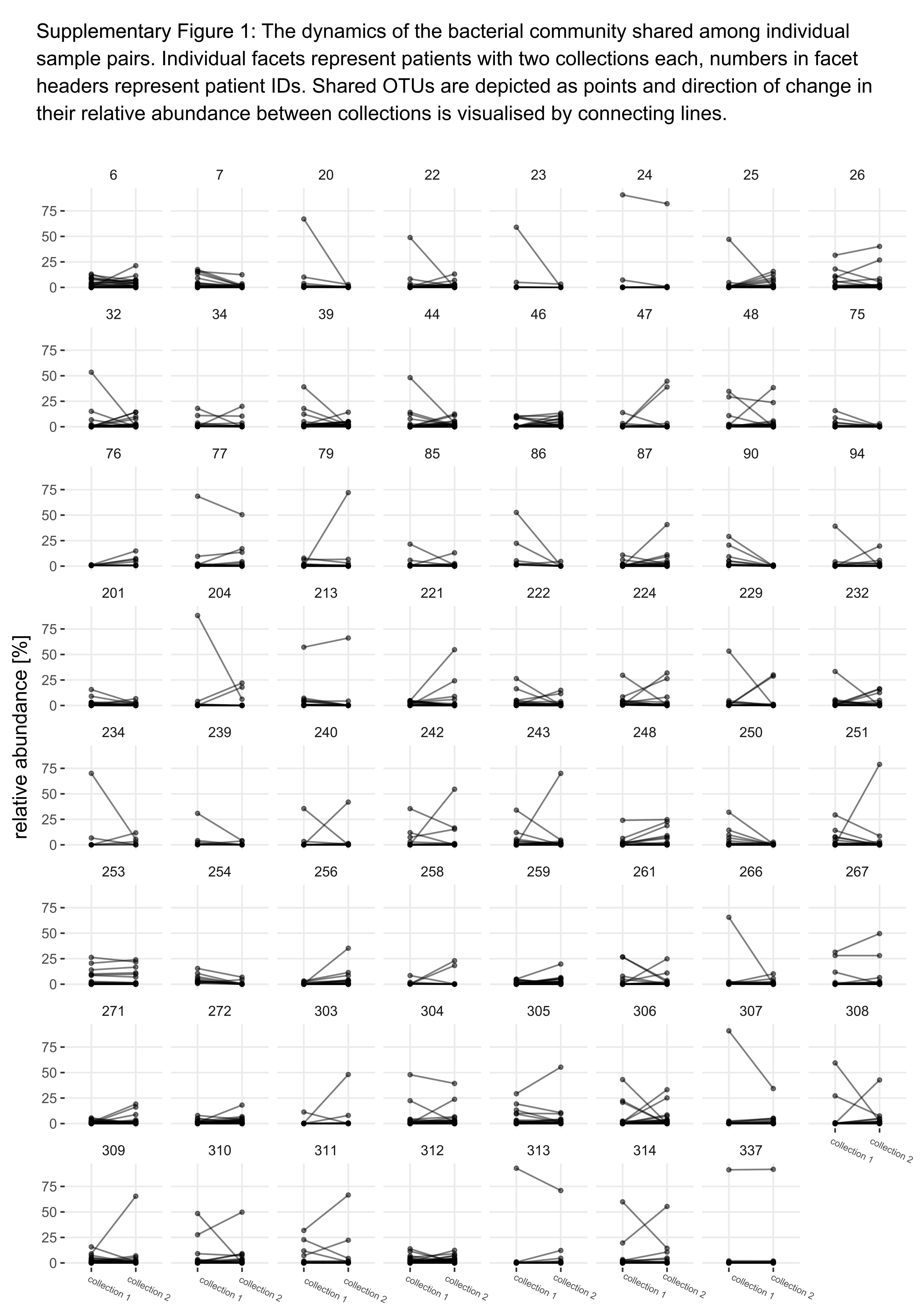

Supplement: Supplementary file 3 — Supplementary Material 3 [file 12866_2025_3915_MOESM3_ESM.tif]
